# Congenital Adrenal Hyperplasia with Salt Wasting Crisis: A Case Report

**DOI:** 10.31729/jnma.4811

**Published:** 2020-01-31

**Authors:** Deepa Khanal, Deependra Mandal, Rajan Phuyal, Uttara Adhikari

**Affiliations:** 1 Department of Pediatrics, Kathmandu Medical College and Teaching Hospital, Sinamangal, Kathmandu, Nepal; 2 Kathmandu Medical College and Teaching Hospital, Sinamangal, Kathmandu, Nepal

**Keywords:** *case reports*, *congenital adrenal hyperplasia*, *steroid 21-hydroxylase*

## Abstract

Congenital Adrenal Hyperplasia is a group of autosomal recessive disorders due to deficiencies of enzymes involved in steroidogenesis. The most common form is a 21-hydroxylase deficiency which can be classical or non-classical. The severe form also called Classical Congenital Adrenal Hyperplasia is usually detected after birth to infant period. If Congenital Adrenal Hyperplasia is not diagnosed and treated early, neonates are susceptible to sudden death in the early weeks of life. We report a case of thirty-five days male with a salt-wasting variant of congenital adrenal hyperplasia. The diagnosis was based on an elevated level of 17-hydroxyprogesterone. He was managed and life long oral Prednisolone and Fludrocortisone were prescribed.

## INTRODUCTION

Congenital Adrenal Hyperplasia (CAH) is an inherited autosomal recessive disorder with a 21-hydroxylase deficiency (21-OHD) due to mutations in the 21-hydroxylase (CYP21A2) gene accounting for 90 percent.^1^ Clinical manifestations depend on the degree of cortisol and aldosterone deficiency. The salt-wasting form can present as a medical emergency with severe hyponatremic dehydration, hyperkalemia with failure to thrive, polyuria and hyperpigmentation.^2^ About 75% of patients having a classical 21-OH deficiency can result in a salt-wasting crisis.^3^ The non-salt wasting can present with clitoral hypertrophy, shallow vagina, the rugosity of labial fold and ambiguous genitalia. Here we report a rare case of congenital adrenal hyperplasia.

## CASE REPORT

A 35-day old male infant presented to our hospital with a history of 3-day diarrhea and vomiting for multiple episodes which were projectile, non-bilious, non blood stained and associated with decreased urinary output. Neither fever nor irritability was noted. He was born at 41^+^4 weeks of gestation by elective cesarean section due to postdated pregnancy with a birth weight of 3.1kg. He cried immediately after birth and was exclusively on breastfeeding till date.

Antenatal scans were normal, maternal serology was non reactive, had no genetic diseases in the family and had no risk factors for sepsis.

On general examination, he was lethargic with sunken eyes, dry oral mucosa, and depressed anterior fontanelle. Vitals were normal. Examinations of the respiratory system, cardiovascular and abdomen were within normal limits. His blood pressure was recorded as 90/50 mm Hg (above 50th centile). His weight was 3.0 kg (z score -2.78), length 55cm (z score -0.67) and head circumference 37 cm (z score 1.78).

**Figure 1.**
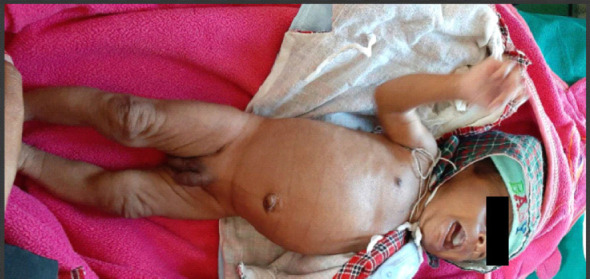
35-day old male with Congenital Adrenal Hyperpplasia showing hyperpigmented genitalia.

On physical examination, normal male genitalia but with pigmentation was noted which was darker than the infant's skin tone (Figure 1).

Investigations showed a negative septic screening with deranged electrolytes. His serum Na^+^ was 116 meq/l, K+ was 6.1meq/l, urea was 33 mg/dl and creatinine was 0.5 mg/dl. Random blood sugar was 75mg/dl. His initial blood gas analysis showed: pH- 7.42 (7.35-7.45), Pco_2_ 23.5 (35-45), HCO_3_ 15.6 mmol/L (22-28 mmol/L), Base excess -9 mmol/L (-4.0 to +4.0 mmol/L), lactate of 2.8 mmol/L (<2 mmol/L). Liver Function Test was within the normal limit.

Both ECG and Ultrasonography findings were normal. Hence, pyloric stenosis was excluded.

A probable diagnosis of CAH with the salt-wasting crisis was made according to examination and investigation and treatment was commenced.

He was given 20ml/kg of 0.9% saline bolus, continuous nebulized Salbutamol (2.5 mg) and Hydrocortisone intravenously (2mg/kg 6 hourly). Blood was drawn and sent for 17-hydroxyprogesterone (17-OHP) which later came to be positive 405ng/ml (<22.0 ng/ml) and confirmed our diagnosis. His glucose and electrolytes have been monitored daily while in the hospital, and they gradually improved by 7th day.

He was discharged home with oral Prednisolone at 15mg/m^2^/day and Fludrocortisone Acetate at 0.3mg/ day. He was followed-up after 8 days in the outpatient department. His weight was 3.5 kg. Electrolytes were normal and medications had been adjusted accordingly based on his weight.

## DISCUSSION

The incidence of classical CAH is 1:10,0001:20,000.^3,4^ In classic 21-hydroxylase deficiency inadequate hydroxylation of progesterone to 11-deoxycorticosterone results in aldosterone deficiency and predispose to a salt-wasting crisis. Other virilizing forms include 3β-hydroxysteroid dehydrogenase and 11β-hydroxylase deficiencies associated with mutations in the 3β-hydroxysteroid dehydrogenase (HSD3B2) and 11β-hydroxylase (CYP11B1) genes, respectively.^5^

Females with classic 21-hydroxylase deficiency are exposed to excess androgens prenatally and are born with virialized external genitalia.^6^ Management of the patient with genital anomalies should consist of a multi-team approach with the active participation of parents. The diagnosis is critical in males due to no genital ambiguity to alert doctors before the onset of dehydration and shock.^7^

Our case presented as an emergency mimicking acute gastroenteritis with diarrhea and vomiting. Since the physical examination in a male child is insufficient to alert a physician for congenital adrenal hyperplasia unlike a female child, the suspicion index was raised only after the lab reports of severe hyponatremia with hyperkalemia and features of dehydration.

Mass screening for 21-hydroxylase deficiency started in Japan in January 1989, and one per 18,000-19,000 infants are found to have a 21-hydroxylase deficiency (OHD).^8^ Many patients with 21-OHD have pigmented skin, masculinisation in the external genitalia (females), poor suckling and low weight gain, but others have 21- OHD with only very mild clinical symptom.

If congenital adrenal hyperplasia is not diagnosed and treated early, neonates are susceptible to sudden death in the first few weeks of life.^9^ The diagnosis is based on hormonal levels of 17-OHP, testosterone, DHEAS, androstenedione, cortisol, andplasma rennin activity.^10^ In our case 17-OHP was elevated. The recognition and appropriate medical management prevented a fatality in this case. The mainstay of treatment is the replacement of glucocorticoid and mineralocorticoid. An increased dose of glucocorticoid is warranted during the period of stress. Our case was managed with normal saline bolus and injection Hydrocortisone and was discharged on oral Prednisolone and Fludrocortisone.

CAH can present as a simple virializing form or life- threatening emergency with severe dehydration. Diagnosing a male infant is particularly difficult owing to an apparently normal physical feature as compared to a female infant with congenital adrenal hyperplasia. Supportive laboratory finding and screening with 17-hydroxyprogesterone is helpful in making a diagnosis and early management of the patient to prevent a fatality.

## References

[1] Speiser PW, White PC (2003). Congenital adrenal hyperplasia. N Engl J Med.

[2] Othman M, Alali N, Aljayar L (2014). Congenital adrenal hyperplasia: Case report. Webmed Central Obstetrics And Gynaecology.

[3] Piaggio LA (2014). Congenital adrenal hyperplasia: review from a surgeon's perspective in the beginning of the twenty-first century. Front Pediatr.

[4] Kriplani A, Lunkad A, Agarwal N, Kulshreshtha B, Ariachery CA (2012). A success story in congenital adrenal hyperplasia. J Obstet Gynaecol India.

[5] Fleming L, Knafl K, Van Riper M (2017). How the child's gender matters for families having a child with congenital adrenal hyperplasia. J Fam Nurs.

[6] Kovacs J, Votava F, Heinze G (2001). Lessons from 30 years of clinical diagnosis and treatment of congenital adrenal hyperplasia in five middle European countries. J Clin Endocrinol Metab.

[7] Canlas JF, Ponmani C (2019). Congenital adrenal hyperplasia with salt-wasting crisis and arrhythmia: a case study. BMJ case Rep.

[8] Ishii T, Anzo M, Adachi M (2015). Guidelines for diagnosis and treatment of 21-hydroxylase deficiency (2014 revision). Clin Pediatr Endocrinol.

[9] Rossignol P, Legrand M, Kosiborod M (2016). Emergency management of severe hyperkalemia: Guideline for best practice and opportunities for the future. Pharmacol Res.

[10] El-Maouche D, Arlt W, Merke DP (2017). Congenital adrenal hyperplasia. Lancet.

